# On Cocaine and Its Hydrochlorate

**Published:** 1885-01

**Authors:** 


ARTICLE VIII.

### ON COCAINE AND ITS HYDROCHLORATE.

Cocaine, the alkaloid of *Erythroxylon Coca* Lam., was discovered by Nieman in 1860, and afterwards studied by W. Lossen (1812,) who assigned to it the formula $C_{17}H_{21}O_4$.

The coca-leaf has been recognized as having qualities somewhat analogous to those of coffee and tea, its alkaloid, however, being entirely different from Caffeine or Theine, which has the composition $C_8H_{10}N_4O_2$. It has lately been brought into especial notice on account of its powers as a local anæsthetic, and there is reason to believe that it will prove a valuable addition to the Materia Medica of the Surgeon and the Dentist.

The coca-leaf is the great source of comfort and enjoyment to the Peruvian Indian; it is to him what betel is to the Hindu, kava to the South Sea Islander, and tobacco to the rest of mankind; but its use produces invigorating effects which are not possessed by the other stimulants. From the most ancient times, the Peruvians have used this beloved leaf, and they still look upon it with feelings of superstitious veneration. In the time of the Yucas it was sacrificed to the sun, the Huillac Umu or high priest chewing the leaf during the ceremony; and before the arrival of the Spaniards, it was used, as the cacao in Mevico, instead of money. After the conquest. although its virtues were extolled by the Yuca Garcilasso de la Vega, and by the Jesuit Acosta, some fanatics proposed to proscribe its use, and to root up the plants because they had been used in the ancient superstitions, and because its cultivation took away the Indians from other work. In 1569, the second Council of Lima, consisting of bishops from all parts of South America, condemned the use of cocoa, because it was a "useless and pernicious leaf, and on account of the belief stated to be entertained by the Indians that the habits of chewing coca gave them strength, which is an illusion of the devil."

In speaking of the strength the coca gives to those who chew it, Garcilasso de la Vega relates the following anecdote: "I remember a story which I heard in my native land of Peru, of a gentleman of rank and honor, named Rodrigo Pantoja, who, traveling from Cuzco to Rimac (Lima,) met a poor Spaniard (for there are poor people there as well as here,) who was going on foot with a little girl, aged two years, on his back. The man was known to Pantoja. and they thus conversed: 'Why do you go laden thus?' said the knight. The poor man answered that he was unable to hire an Indian to carry the child, and for that reason he carried her himself. While he spoke, Pantoja looked in his mouth and saw that it was full of coca, and as the Spaniards abominate all that the Indians eat and drink, as though it savored of idolatry, particularly the chewing of coca, which seems to them a low and vile habit, he said: 'It may be as you say, but why do you eat coca like an Indian, a thing so hateful to Spaniards?' The man answered, 'In truth, my lord, I detest it as much as any one, but necessity obliges me to imitate the Indians and keep coca in my mouth, for I would have you know that if I did not do so I could not carry this burden, while the coca gives me sufficient strength to endure the fatigue.' Pantoja was astonished to hear this, and told the story wherever he went, and from that time credit was given to the Indians for using coca from necessity and not from vicious gluttony."

The coca plant (*Erythroxylon coca*) is cultivated between 5,000 and 6,000 feet above the level of the sea, in the warm valleys of the Eastern slopes of the Andes, where almost the only variation of climate is from wet to dry, where frost is unknown, and where it rains more or less every month in the year. It is a shrub, from four to six feet high, with lichens, called *lacco* in uichua' usually growing on the older trunks. The branches are straight and alternate and entire, in form and size, like tea-leaves; flowers solitary, with a small yellowish white corolla, in fine petals, ten filaments the length of the corolla, anthers heart-shaped, and three pistils.

Sowing is commenced in December and January, when the rains begin, which continue until April. The seeds are spread on the surface of the soil in a small nursery or raising ground, called *almaciga*, over which there is generally a thatch roof (*huaschi.*) At the end of about a fort-night they come up; the young plants being continually watered, and protected from the sun by the hausichi. The following year they are transplanted to a soil specially prepared by thorough weeding and breaking up the clods very finely by hand, often in terraces, only affording room for a single row of plan;s, up the sides of the mountains, which are kept up by small stone walls.

At the end of eighteen months the plants yield first harvest, and continue to yield for upwards of forty years. The green leaves called *matu* are deposited in a piece of cloth which picker carries, and are then spread out in the drying-yard, called *matu-concha*, and carefully dried in the sun. The dried leaf is called *coca*. The greatest care is required in the drying; for too much sun causes the leaves to dry up and lose their flavor, while if packed up moist they become fetid. They are generally exposed to the sun in thin layers.

The approximate annual produce of coca in Peru is about 15,000,000 lbs., the average yield being about 800 lbs. an acre. More than 10,000,000 lbs. are produced annually in Bolivia; so that the annual yield of coca throughout South America, including Peru, Bolivia, Ecuador, and Pasto, may be estimated at more than 30,000,000 lbs. At Tacna, the *tambor* of 50 lbs. is worth nine to twelve dollars, the fluctuations in price being caused by the perishable nature of the article, which cannot be kept in stock for any length of time. *The average duration of coca in a sound state, on the coast, is about five months*, after which time it is said to lose flavor, and is rejected by the Indians as worthless.

The smell of the leaf is agreeable and aromatic, and when chewed it gives out a grateful fragrance, accompanied by a slight irritation, which excites the saliva. Its properties are to enable a greater amount of fatigue to be borne with less nourishment, and to prevent the occurance of difficulty of respiration in ascending steep mountain sides. Tea made from the leaves has much the taste of green tea, and if taken at night is much more effectual in keeping people awake. Applied externally, coca moderates the rheumatic pains caused by cold, and cures headaches. When used to excess it is, like everything else, prejudicial to the health, yet, of all the narcotics used by man, coca is the least injurious and the most soothing and invigorating.

Its qualities ought to recommend its use to members of the Alpine Club, and to walking tourists in general, though the deterioration of Coca leaves in transportation, especially in sea voyages, renders the quality of different samples exceeding variable. The alkaloid forms but a small proportion of the mass at best, and in poor samples is very scanty.

Lossen obtained, in the most favorable case, 4 parts per 1,000, and from poor material only 1.6 parts per 1,000.

Cocaine crystallizes in four or six-sided monoclynic prisms. It is soluble at 12° C. in 704 parts of water; easily soluble in alcohol, and still more so in ether. It melts near 92°.

Cocaine combines easily with dilute acids, forming easily cyrstallizable salts, which are soluble in alcohol, but *insoluble in ether*, have a bitter taste, and leave a transient sensation of insensibility upon the tongue.

Hydrochlorate of cocaine, $C_{17}H_{21}NO_4HCI$, separates from its aqueous solution in short transparent, prismatic crystals, which are permanent in the air.

Acetic acid dissolves cocaine readily, but on evaporation the base separates again in crystals. Niemann took these for the acetate.

Nitrate of cocaine crystallizes with great difficulty.

Neutral sulphate of cocaine is a transparent gummy mass, becoming only slowly crystalline.

Schroff, who made the first experiments with cocaine in 1862, observed that it caused fluctuation in the respiration and pulse, and produced mydriasis. Fronmuller (1863) found that it had but little effect in man, in doses of 0.03 to 0.33 Gm. (two-fifths to one-half grain,) and in the case of an attempted suicide, 1.5 gm. (24 grains) did not even produce serious results. The fatal dose, therefore, seems to be much higher, unless the cocaine used in those days was very impure.

According to Merck, coca contains between one-fifth and one-fifty per cent. of cocaine. The average effective dose of the hydrochlorate, in man, is stated to be 0.95 gm. (¾ grain.)

Last year cocaine was recommended as a remedy in cases of great exhaustion from various causes. Its use in such cases suggested itself naturally from the well-known fact that coca has long been used as a preventive of the waste of tissues, fulfilling the same function as tea, coffee, and other substances of this nature. It has also been found of great value in the treatment of morphinism, supplying the craving for stimulants resulting from the gradual or sudden withdrawal of morphine or opium. Dr. Freud has reported very favorable results in morphinism; when morphine is to be slowly withdrawn, the dose is gradually lowered, and that of cocaine proportionately increased. Dr. Freud reports complete recovery in one case after ten days' treatment, and believes that there is a positive antagonism between morphine and cocaine.

It has been often noticed by different experimenters since 1863, that cocaine produces temporary insensibility or numbness when applied to certain mucous membranes, but no practical use suggested itself until quite recently. In the United States it has been used to a considerable extent to lessen the irritability of the soft palate and pharynx in laryngoscopic examinations.

Dr. Koller, of Viena, the discoverer of the anæsthetic effect of the remedy, found that immediately after the instillation of a two per cent. solution of hydrochlorate of cocaine into the eye, there was felt a short feeling of burning lasting for about one-half minute, which was soon followed by an indistinct feeling of dryness. The aperture of the eyelids appears wider, and reflex actions (such as are usual when the cornea is touched: twitching of the head, of the lids, turning aside of the eyeball, etc.,) disappear. When this condition prevails, pressure may be made upon the cornea or a portion of the conjunctiva be caught with pincers, without any disagreeable sensation being felt. The anæsthesia of the eye lasts for about ten minutes, and a low degree of sensibility persists for several hours. Twenty to thirty minutes after the application, the pupil becomes dilated, returning in about twelve hours to its normal condition. The only abnormal symptom observed during this condition is a slight paralysis of accommodation. which can, however, be overcome by a little effort.

Since Dr. Koller publicly exhibited his experiments and prophesied for cocaine a speedy general use in ophthalmological practice (for operations, etc,) the new drug has been experimented with in all countries, the United States included, and those who have used it and seen its operation are quite enthusiastic over it. Moreover, it has already been ascertained that it acts as an anæsthetic not only on the conjunctiva of the eye, but on all mucous membranes.

Prof. W. M. Polk, M. D., of New York, has used a four per cent. solution of cocaine as an anæsthetic in an operation for abscess of the liver, the solution being applied to the open wound; and under its anæsthetic effect the operation was completed within twenty-five minutes without any discomfort to the patient.

On October 29, 1884, Dr. Polk closed rents of the cervix uteri in two cases, after applying the solution of cocaine as given above. Three applications were made two or three minutes apart, and the operation was commenced about three minutes after the last application. The first operation lasted forty minutes, but no complaint was made until the last ten minutes, and then the discomfort was a sense of soreness rather than acute pain. In the second case, there was more sensitiveness to the touch before the application of the solution. No pain was felt, however, until after the lapse of twenty minutes, when it was necessary to re-apply the solution, which was done directly to the cut surfaces, after freeing them from blood.

The testimony of this patient is valuable, as she had previously undergone the same operation with ether, and she unhesitatingly preferred the application of cocaine.

Dr. J. R. Uhler, of Baltimore, writes to the Maryland *Medical Journal*, that the application of the solution of the muriate of cocaine to the vulva mitigates the pains of labor.

Dr. Felix Semon, of London, England, has used it successfully in intra-larnygeal operations.

Dr. C. Bader, of Guys Hospital, has repeatedly used it in operations on the eye.

Dr. E. H. Bosworth reports, in the *Medical Record* (Nov. 15th,) that on applying a solution of cocaine (2 per cent.) to the nasal passages, the venous sinuses below the muccus membrane become, within twenty or thirty seconds so rigidly contracted as to expel all the blood contained in them, and to cause the membrane to cling closely to the bony structure which then becomes sharply outlined. He has used the drug in hypertraphy of the nasal mucous membrane (*nasal catarrh*,) acute *coryza* and in operations for nasal *polypus*. In each case the venous congestion or turgescence was so thoroughly kept down that all discomfort was removed, and in the case of polypi not only the recognition and removal of the growth became quite easy, but also turned out a bloodless operation.

Dr. Bosworth also suggests the use of cocaine in hay-fever.

A four-per-cent solution is now generally preferred when prompt action is required.

We may add that the drug appears to be of value particularly also to dentists. While its effects may be too superficial to render it applicable in cases of extraction, it will undoubtedly add much to the patient's comfort when ligatures are to be forced under the gum in the application of the rubber dam, and it may possibly be of service as an alleviator of sensitive dentine. Its topical application will probably be found in some forms of neuralgia, and in odontalgia. Cases of pulpitis will probably be relieved, on account of its property of relieving venous congestion, as mentioned by Dr. Bosworth (*supra*).

To produce local anæsthesia it is only necessary to paint the part with the solution two or three times at intervals of as many minutes. Insensibility of the part will supervene in about five minutes, and continue from twenty to thirty minutes. It may also be injected hypodermically with good effect. A two or four-per-cent. solution is generally employed, but a stronger one is sometimes required, even as strong as twenty per cent.

If ever a remedy, on purely theoretical grounds, held out any hopes of being serviceable in sea-sickness, it seems to us that cocaine is one of these. At all events, it will be worth while to tr its effects.

We are indebted to the below-named journals for the data for the preceeding article:

*American Druggist*, Dec. 1884; *The Lancet*, Nov. 22, 1884; *N. Y. Medical Record*. Nov. 15, 1884; *Buffalo Medical and Surgical Journal*, Dec. 1884; *The Dental Advertiser*, Jan. 1885.

